# A multiplex immunoassay of serum biomarkers for the detection of uveal melanoma

**DOI:** 10.1186/s12014-019-9230-8

**Published:** 2019-03-05

**Authors:** Jin Song, Shannath L. Merbs, Lori J. Sokoll, Daniel W. Chan, Zhen Zhang

**Affiliations:** 1 Center for Biomarker Discovery and Translation, Department of Pathology, Johns Hopkins University School of Medicine, Baltimore, MD 21287 USA; 2 Department of Ophthalmology, Johns Hopkins University School of Medicine, Baltimore, MD 21231 USA; 3 Department of Pathology, Johns Hopkins University School of Medicine, 419 North Caroline Street, Baltimore, MD 21231 USA

**Keywords:** Multiplex, Immunoassay, Serum, Biomarker, Uveal melanoma

## Abstract

**Background:**

Approximately 50% of uveal melanoma (UM) patients develop metastases preferentially in the liver leading to death within 15 months. Currently, there is no effective treatment for metastatic UM, in part because the tumor burden is typically high when liver metastases are detected through abnormal liver function tests (LFTs) or imaging studies. The use of LFTs results followed by diagnostic tests has high specificity and predictive values but low sensitivity, and better tests are needed for early diagnosis of the primary tumor as well as its metastatic spread. To evaluate serum biomarkers for the early detection of UM, multiplex immunoassays were developed.

**Methods:**

Magnetic bead-based multiplex immunoassays were developed for the selected serum biomarkers using a Bio-Plex 200 system. The dynamic ranges, lower limits of detection and quantification, cross-reactivity, and intra- and inter-assay precision were assessed. All proteins were analyzed in sera of 48 patients diagnosed with UM (14 metastatic, 9 disease–free (DF) ≥ 5 years, 25 unknown) and 36 healthy controls. The performance of the biomarkers was evaluated individually and in combination for their ability to detect UM.

**Results:**

A 7-plex immunoassay of OPN, MIA, CEACAM-1, MIC-1, SPON1, POSTN and HSP27 was developed with negligible cross-reactivity, recovery of 84–105%, and intra-assay and inter-assay precision of 2.3–7.5% or 2.8–20.8%, respectively. Logistic regression identified a two-marker panel of HSP27 and OPN that significantly improved the individual biomarker performance in discriminating UM from healthy controls. The improved discrimination of a two-marker panel of MIA and MIC-1 was also observed between metastatic UM and DF, however not statistically significant due to the small sample size.

**Conclusions:**

The multiplex immunoassay provides sufficient analytical performance to evaluate serum biomarkers that complement each other in detection of UM, and warrants further validation with a larger number of patient samples.

**Electronic supplementary material:**

The online version of this article (10.1186/s12014-019-9230-8) contains supplementary material, which is available to authorized users.

## Background

Uveal melanoma (UM) is the most common primary intraocular malignant tumor in adults. It affects the uveal track of the eye, namely, the iris, ciliary body, and the choroid, with 90% of the cases involving the choroid [1]. The overall incidence is approximately 5–7 per million per year in the United States [2, 3], with a 5-year mortality rate of up to 30% [4, 5]. Despite fewer than 1% of patients presenting with clinical evidence of distant metastasis at the time of treatment for their intraocular lesion, they carry a risk of disease recurrence even years after control of the primary tumor. Approximately 50% of patients ultimately develop fatal metastases primarily in the liver via a hematogenous route leading to death mostly within 15 months [2, 6, 7]. Currently, there is no effective therapy for metastatic UM because the tumor burden from metastases to liver is typically high by the time of detection through abnormal liver function tests (LFTs) or imaging studies [8–10]. LFTs, including those for lactate dehydrogenase (LDH), alkaline phosphatase (ALK-P), aspartate aminotransferase (AST), alanine aminotransferase (ALT), γ-glutamyl transpeptidase (γ -GT), and total bilirubin (TBil), are widely used clinically for detecting metastasis of UM, but lack the necessary sensitivity and specificity [11]. Appropriate radiographic surveillance for UM patients following treatment of their primary lesion is also unclear. The Collaborative Ocular Melanoma Study (COMS) concluded that the use of LFTs results followed by diagnostic tests has high specificity and predictive values but low sensitivity, and better tests are needed to identify earlier metastatic disease associated with choroidal melanoma [12]. Micrometastasis may be present in majority of patients at the time of initial diagnosis, but undetectable by conventional methods due to their small size and reduced cell turnover. Therefore, there is an urgent need to develop sensitive biomarkers for early diagnosis of the primary tumor as well as its metastatic spread.

Some proteins synthesized, secreted or shed into the blood, have been shown to be promising serum biomarkers in detecting metastatic UM [13]. Melanoma inhibitory activity (MIA), S100 calcium binding protein B (S-100B), osteopontin (OPN), tissue polypeptide-specific antigen cytokeratin 18 (TSP), macrophage inhibitory cytokine-1 (MIC-1), parkinson protein (DJ-1/PARK7), and soluble hepatocyte growth factor receptor (c-Met) were significantly elevated in serum of metastatic UM patients when compared with nonmetastatic patients [9, 14–25]. However, the wide interindividual variability of serum biomarkers such as vascular endothelial growth factor (VEGF) precluded the use of any cut-off level to determine the metastatic status of an individual UM patient based on a single biomarker serum level, even though the serum biomarker increased significantly after metastases developed [26]. When used in combination, serum OPN, MIA and S100B provided a highly sensitive method to detect liver metastasis of UM with an area under the curve (AUC) of 91%, which was far superior to LFTs [14]. Despite better predictions with combinations of multiple serum biomarkers, these markers have not proven to be robust predictors of metastatic disease nor have they been adopted into universal clinical practice.

Additional potential biomarkers were recently reported to have an association with the progression of UM and other malignancies using different technical platforms [13, 27, 28]. It was reported that serum carcinoembryonic antigen cell adhesion molecule-1 (CEACAM-1) correlated with disease progression and survival in malignant cutaneous melanoma patients [29]. CEACAM-1 was also found expressed in both primary and metastatic UM through an immunohistochemical evaluation, and correlated with poor prognostic factors such as epithelioid cell type and networks of extracellular matrix pattern [30]. Heat shock protein 27 (HSP27) was associated with a poor prognosis in gastric, liver, prostate, lung, and breast cancer [31]. But, its expression was shown to be significantly lower in monosomy 3 uveal melanoma when compared with disomy 3 tumors by immunohistochemistry [32, 33]. Upregulation of periostin (POSTN) has been observed in many cancer types, such as ovarian, pancreatic, breast, and bladder cancer [34]. POSTN also accelerated human malignant cutaneous melanoma progression by modifying the melanoma microenviroment [35]. Spondin 1 (SPON1) has been identified as a potential biomarker of ovarian cancer [36], and also downregulated during cutaneous melanoma development [37].

Novel candidate biomarkers identified using different high-throughput proteomic technologies along with other potential biomarkers reported in the literature need to be rigorously validated. Enzyme-linked immunosorbent assays (ELISAs) are widely used to quantify substances such as peptides, proteins, antibodies, and hormones in plasma or serum, and can be highly sensitive, specific, precise and accurate methods with low detection limits, high user-friendliness and robustness. However, traditional ELISAs only measure a single antigen at a time, which can be a major challenge for simultaneously quantification of multiple potential biomarkers across large cohorts of patient samples. Magnetic bead-based multiplex immunoassays represent a promising solution that simultaneously measures multiple analytes in a single sample using minimumal sample volume. The use of differentially detectable bead sets as substrates capturing analytes in solution and detection antibodies measuring quantities of analytes enables the simultaneous identification and quantification of many analytes in the same sample. Compared with traditional ELISA and planar microarray, magnetic bead-based immunoassays may demonstrate faster solution-phase kinetics compared to solid-phase kinetics resulting in lower limits of quantification [38, 39]. To evaluate serum biomarkers for the detection of UM, a 7-plex immunoassay of OPN, MIA, CEACAM-1, MIC-1, SPON1, POSTN and HSP27 was developed as described previously [40], and applied to a set of serum samples from UM patients and healthy controls. The performance of the biomarkers was assessed individually and in combination in their ability to detect UM.

## Methods

### Patient specimens

A total of 84 archived serum samples obtained from 48 patients with UM from May 2004 to February 2014 and 36 healthy controls from either February 2005 or April 2013 were collected at the Johns Hopkins Medical Institutions (JHMI) with institutional approval. Among 48 patients with UM, 14 died with clinically overt metastases from UM, 9 were disease-free (DF) for at least 5 years following treatment of the primary tumor, and 25 were either with shorter surveillance follow-up or relevant clinicopathologic information not available. Detailed clinicopathologic characteristics of the study cohort, including age, sex, cell type and metastasis status, are shown in Table 1. All patient serum samples were obtained before treatment and before surgery, and stored at -80ºC until analysis.

**Table 1:** Clinicopathologic characteristics of the study cohort

Variables	Number (%)
Total	84
Healthy control	36 (42.9)
Age (years)	
Mean ± SD	45.4 ± 14.0
Range	24–78
Gender	
Male	10 (27.8)
Female	26 (72.2)
Uveal Melanoma	48 (57.1)
Age (years)*	
Mean ± SD	61.8 ± 13.6
Range	25–88
Gender	
Male	29 (60.4)
Female	17 (35.4)
NA	2 (4.2)
Cell type	
Spindle	8 (16.7)
Mixed	25 (52.1)
Epithelioid	5 (10.4)
NA	10 (20.8)
Metastasis status	
Met	14 (29.2)
DF	9 (18.8)
NA	25 (52.1)

NA, relevant clinicopathologic information not available or shorter surveillance follow-up. Met, patients who died with clinically overt metastases from uveal melanoma. DF, patients who were disease-free for at least 5 years following treatment of the primary tumor

*2 patients without relevant information

### Reagents and antibodies

All the recombinant proteins and antibodies were purchased from R&D Systems (Minneapolis, MN)), except the detection antibody for SPON1 which was biotinylated in-house. Majority of the antibodies except those for OPN and SPON1 were from the DuoSet ELISA kits (R&D). Detailed information for the recombinant proteins and antibodies is shown in Additional file 1. Magnetic COOH beads, amine coupling kits, and Bio-Plex Pro Reagent kits were purchased from Bio-Rad Laboratories (Hercules, CA). NHS and Sulfo-NHS, EDC, EZ-Link™ Sulfo-NHS-Biotin, and Zeba™ Spin Desalting Columns were purchased from Thermo Scientific (Rockford, IL).

### Conjugation of antibodies to microspheres

The capture antibodies for OPN, MIA, CEACAM-1, MIC-1, SPON1, POSTN and HSP27 were coupled to magnetic beads of different regions using the Bio-Rad amine coupling kit according to the manufacturer’s instructions. The use of differentially detectable beads of different regions enables the simultaneous identification and quantification of multiple analytes in the same sample allowing the individual immunoassays to be multiplexed. The optimal amounts of capture antibodies for one coupling reaction were used at 6 µg for OPN, MIA, CEACAM-1, MIC-1, and HSP27 and 9 µg for SPON1 and POSTN following titration. The coupled beads were counted using a Coulter Z2 counter, validated using biotinylated rabbit anti-mouse (B8520) or rabbit anti-goat (B7014) IgG antibodies (Sigma-Aldrich, St. Louis, MO), and stored in storage buffer at 4ºC in the dark.

### Multiplex immunoassay

**Fig. 1:**
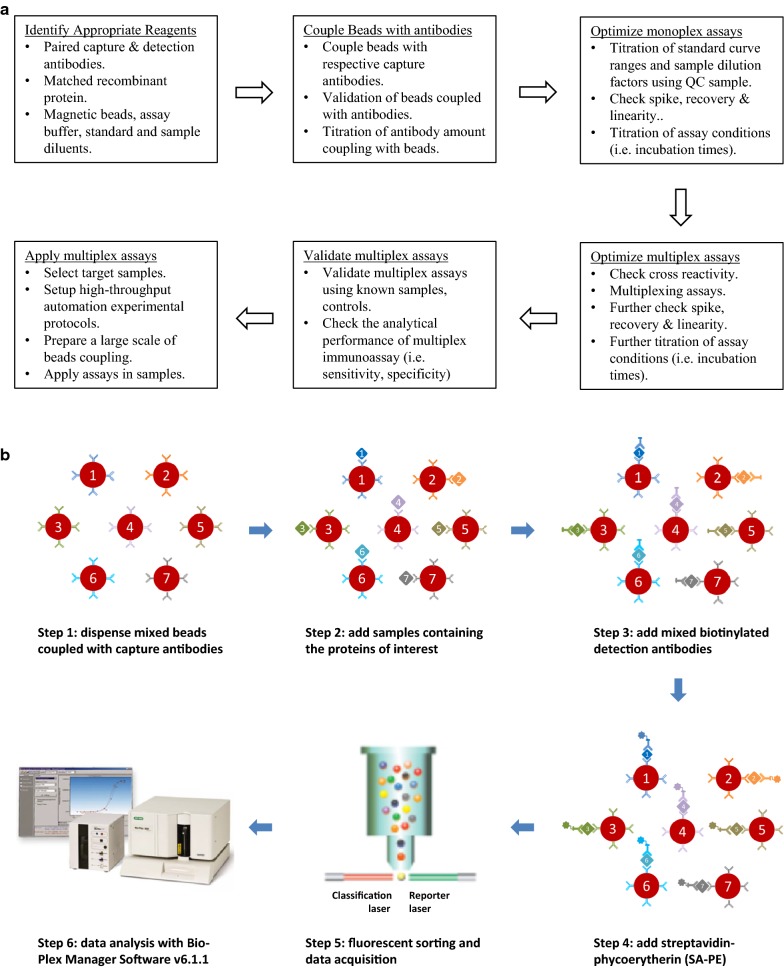
The general view of magnetic bead-based multiplex immunoassay development and application. **a** Flowchart of multiplex immunoassay development and application. **b** Workflow for magnetic bead-based multiplex immunoassay

The magnetic bead-based multiplex immunoassay was developed for the selected candidate serum biomarkers using a Bio-Plex 200 suspension array system (Bio-Rad, Hercules, CA). The general workflow of multiplex immunoassay is shown in Fig. 1. The monoplex immunoassays of individual candidates were first developed using the Bio-Plex Pro Reagent kit. Briefly, 2500 coupled beads were incubated with 50 µl of a sample diluted in sample diluent for 1 h. The beads were washed and incubated with 25 µl of the detection antibody diluted in the detection antibody diluent for 30 min. The beads were then washed again and incubated with 50 µl of 2 µg/mL streptavidin-phycoerytherin (SA-PE) diluted in the assay buffer for 10 min. The beads washed a final time and suspended in 125 µl of the assay buffer for the analysis with the Bio-Plex 200 system. All assays were carried out at room temperature and protected from light. All washing steps were performed with the washing buffer with an automated plate washer (Bio-Plex Pro™ II wash station, Bio-Rad). Calibration curves were established using 9 calibrators in 2-fold dilution series and used to determine the protein concentrations. Two pooled human normal sera (one internal pooled sera and the other S7023 from Sigma-Aldrich) were used for optimization of assay conditions.

Prior to multiplexing the individual assays, assay specificity was examined by performing single-antigen and single-detection antibody cross-reactivity studies to detect the fluorescence signals in response to high concentrations of the recombinant proteins at least at the third dilution point of the standard curve. The single-antigen study was conducted by testing an individual antigen in the presence of multiplexed capture beads and detection antibodies, which evaluates the specificity of a capture antibody. The single detection antibody study was conducted by testing an individual detection antibody in the presence of multiplexed capture beads and antigens, which evaluates the specificity of a detection antibody and to some degree the specificity of the capture antibody. Cross-reactivity was defined as the percentage of nonspecific cross-reacting signal detected relative to the specific signal for that analyte.

For the multiplex immunoassay, the capture beads and the detection antibodies were prepared by mixing the 2500 coupled beads and the detection antibodies used in the monoplex assays. The final concentrations of the detection antibodies in the multiplex assay were used at 0.4 µg/mL for OPN, CEACAM-1 and HSP27; 2 µg/mL for SPON1 and POSTN; 0.2 µg/mL for MIA; and 0.05 µg/mL for MIC-1, after the titration. The calibration curve was established using 9 calibrators in 2-fold dilution series derived from a mixture of the highest standard points of 7 recombinant proteins. The highest standards of the 7 recombinant proteins in the multiplex assay were used at 40, 2, 30, 12, 24, 25 and 2 ng/mL for OPN, MIA, CEACAM-1, MIC-1, SPON1, POSTN and HSP27, respectively. To assess the correlations of the developed immunoassays for protein quantification, the multiplex immunoassays were compared to the monoplex immunoassays by measuring 4 independent doses of individual recombinant proteins spiked in the same sample diluent based on their respective calibration curves. The correlations of the developed multiplex immunoassays and commercial ELISA kits in serum OPN, HSP27 and POSTN protein quantifications were also determined in 6, 13 or 7 human sera, respectively. The multiplex immunoassay was carried out using the Bio-Plex Pro Reagent kit with the same procedures as those in the monoplex assays described above. The serum samples were diluted 4-fold in the sample diluent in the multiplex immunoassay. Two quality controls (QC) were prepared by diluting the mixture of the highest standards of 7 recombinant proteins at either 3-fold (QC1) or 30-fold (QC2). The multiplex immunoassay was performed in triplicate on 4 × 96-well Bio-Plex flat bottom plates with a calibration curve and 2 QCs in each plate. All samples were randomized with regard to their plate locations.

Data acquisition and primary data analysis were performed on the Bio-Plex 200 system in combination with Bio-Plex Manager Software version 6.1.1 by use of a 5-parametric (5-PL) nonlinear logistic regression curve fitting model (Bio-Rad). According to Bio-Rad Bio-Plex multiplex immunoassay handout (http://www.bio-rad.com/en-us/applications-technologies/bio-plex-multiplex-immunoassays), in this study, the assay sensitivity (limit of black, LOB) was defined as the concentration of analyte corresponding to the median fluorescent intensity (MFI) of the background plus two standard deviations (SD) of the mean background MFI. The assay reproducibility was assessed in both intra- and inter-assay precisions. Intra-assay precision was calculated as the coefficient of variance (%CV) on the triplicates of two QCs on a single assay plate. Inter-assay precision was calculated as the %CV from 4 independent assays. The assay recovery was calculated as the percentage of the observed concentration relative to the expected concentration of each standard point or QC. The assay working dynamic range was defined as the range between the lower limit of quantification (LLOQ) and the upper limit of quantification (ULOQ) in which an assay is both precise (intra-assay  %CV ≤ 10% and inter-assay  %CV ≤ 15%) and accurate (80–120% recovery).

### Data analysis

The nonparametric Mann–Whitney U test was used to compare serum biomarker levels between UM patients and healthy controls or different subgroups of UM patients, with a *p* value less than 0.05 considered significant. Receiver-operating-characteristic (ROC) curve analysis was performed and the area under the curve (AUC) was calculated separately for each of 7 biomarkers and the combinations of biomarkers. Delong test was used to compare the AUCs. Pearson correlation coefficients were determined to assess correlation of the measurements between the multiplex and monoplex immunoassays or commercial ELISA kits. Logistic regression modelling was constructed including age and sex as covariates and forward stepwise selected log transformed variables with the highest performance. The Statistica 13.3 (StatSoft), GraphPad Prism 6 (GraphPad Software), and MedCalc version 18.10.2 (MedCalc Software bvba) were used for statistical analysis.

## Results

### Development and validation of a 7-plex immunoassay

Customized magnetic bead-based multiplex immunoassays were developed for the selected candidate serum biomarkers using a Bio-Plex 200 suspension array system. Comprehensive literature searching and in silico analysis of publicly available gene and protein databases were performed to identify numerous biomarker candidates which have been reported to be involved in the progression of melanoma and also measureable in human serum. The final candidates were selected for the multiplex immunoassay development based on the commercial availability of appropriate pairs of capture and detection antibodies and their relative abundances in human serum samples. Magnetic bead-based monoplex immunoassays were first developed for OPN, MIA, CEACAM-1, MIC-1, SPON1, POSTN and HSP27 using pooled human normal sera. The cross-reactivity studies through single-antigen and single-detection antibody experiments indicated that the degree of cross-reactivity across the 7 immunoassays was generally < 2%, based on the measurements in response to high concentrations of the recombinant proteins at least at the third dilution point of the standard curve. Approximately 2.4–6.2% of nonspecific cross-reactivity as observed in POSTN or HSP27 antibodies against other proteins (data not shown). But, it should be noted that majority of this nonspecific cross-reactivity was observed at recombinant protein concentrations that exceed physiological levels, thereby reducing the chance of cross-reactivity.

**Fig. 2:**
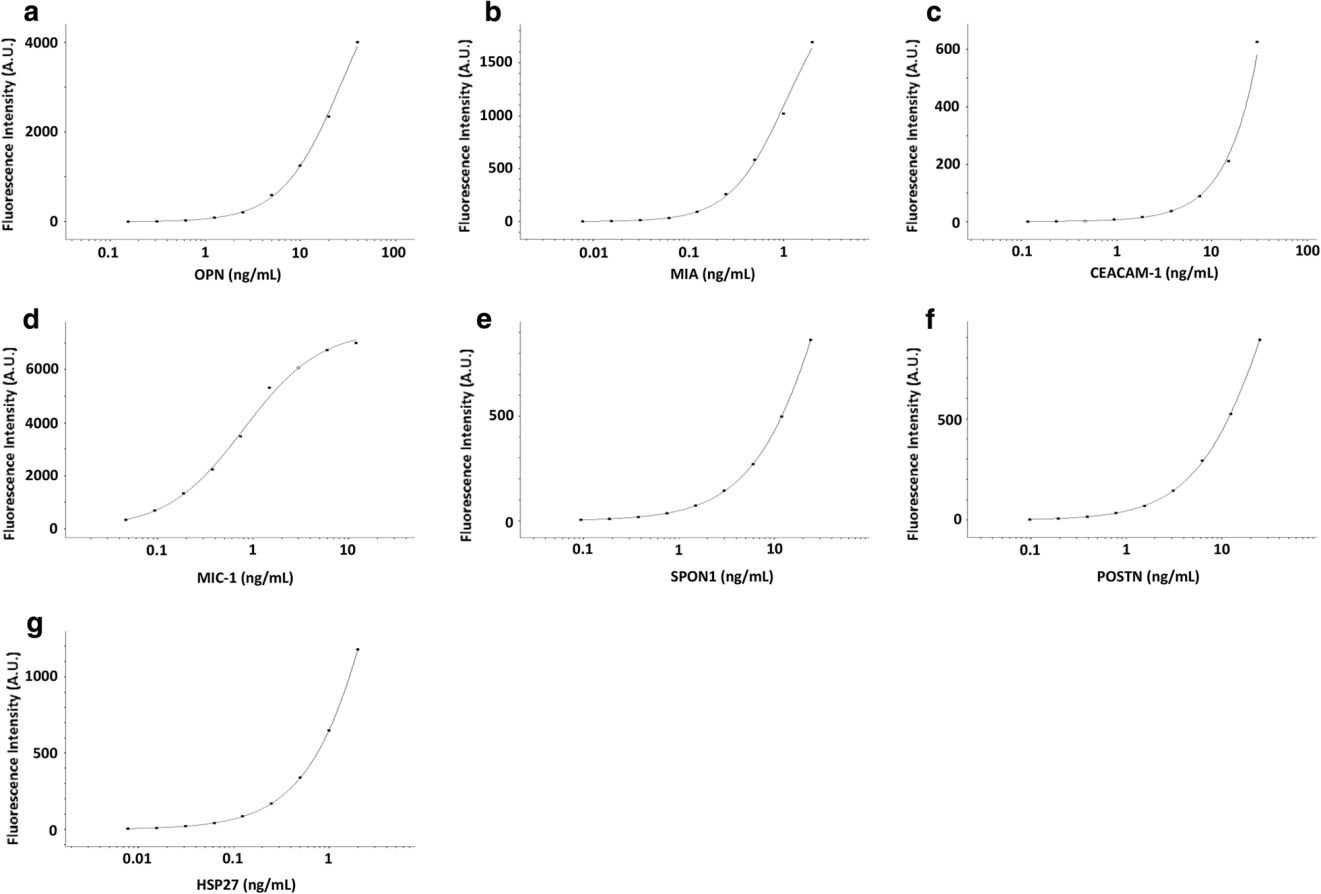
Calibration curves of the 7-plex immunoassay. **a**–**g** calibration curves of OPN, MIA, CEACAM-1, MIC-1, SPON1, POSTN and HSP27 in the 7-plex immunoassay generated using the 5 parameter (5PL) logistic regression model. A.U., arbitrary units

**Fig. 3:**
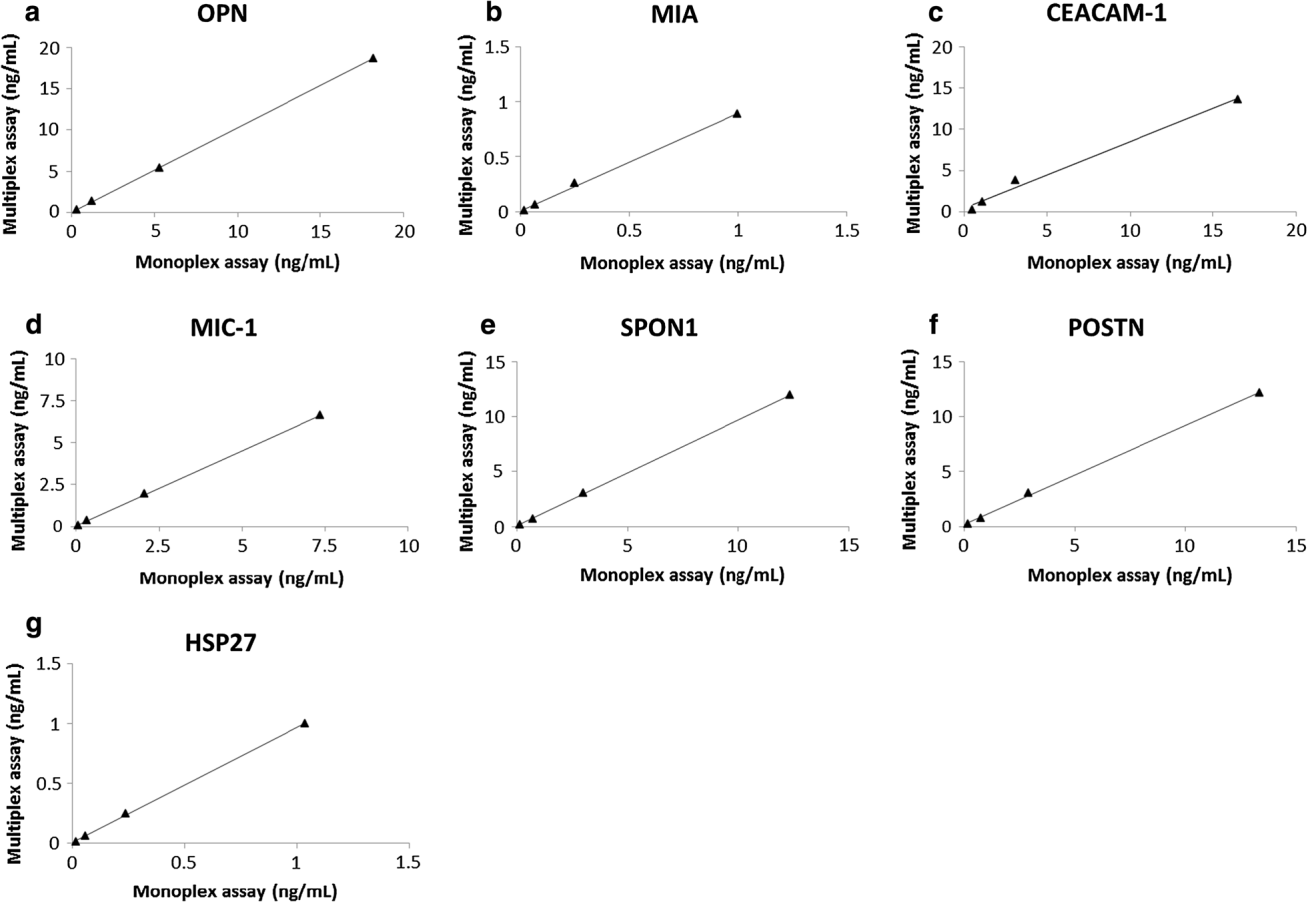
Comparison of the multiplex immunoassay and monoplex immunoassay. **a**–**g** correlations of the 7-plex immunoassay and their respective monoplex immunoassays for measurement of OPN, MIA, CEACAM-1, MIC-1, SPON1, POSTN and HSP27

By mixing the capture antibody-coupled beads and detection antibodies used in the monoplex immunoassays, a 7-plex immunoassay of OPN, MIA, CEACAM-1, MIC-1, SPON1, POSTN and HSP27 was developed and evaluated. The calibration curves of the 7-plex immunoassay generated using the 5PL logistic regression models are shown in Fig. 2a–g. The 7-plex immunoassay results significantly correlated with their respective monoplex immunoassay results (Fig. 3a–g; all Pearson R = 0.99 and *p *< 0.006), suggesting that the 7-plex immunoassay was comparable to the monoplex immunoassays for protein quantification. The 7-plex immunoassay results also correlated significantly with commercial ELISA measurements of OPN (Pearson R/*p* value, 0.81/0.0487), HSP27 (0.91/0.00002), and POSTN (0.90/0.0064). To evaluate the possible effects of high background due to POSTN antibody clones (R&D), the 7-plex immunoassay were also compared to the 6-plex immunoassay withtout POSTN for protein quantification in 8 human sera. The 7-plex immunoassay results were found to be significantly correlated with the 6-plex immunoassay results of serum OPN (Pearson R/*p* value, 0.91/0.0015), MIA (0.88/0.0038), CEACAM-1 (0.87/0.0046), MIC-1 (0.94/0.0007), SPON1 (0.90/0.0024), and HSP27 (0.93/0.0007) levels.

The analytical performance of the 7-plex immunoassay is shown in Table 2, with recovery of 84–105% (standard curve points and QCs), intra-assay precision of 2.3–7.5% (QCs), and inter-assay precision of 2.8–20.8% (QCs). The 7-plex immunoassay exhibited wide dynamic concentration ranges (> 2 log) defined by LLOQ and ULOQ and low LOBs for target protein quantification.

**Table 2:** Analytical performance of the 7-plex immunoassay

	Mean (pg/mL)		Intra-assay Precision (%CV)		Inter-assay Precision (%CV)		LOB (pg/mL)	LLOQ (pg/mL)	ULOQ (pg/mL)	Triplicates* (%CV)
	QC1	QC2	QC1	QC2	QC1	QC2				
OPN	13650.0	1435.0	4.4	6.8	5.2	14.5	278.8	389.5	40024.5	7.1
MIA	670.0	67.5	2.3	3.9	7.0	14.2	12.5	16.3	1999.0	4.7
CEACAM-1	9540.0	967.5	7.5	7.4	5.4	20.8	351.2	548.5	30079.5	5.0
MIC-1	2977.5	297.5	3.0	3.6	3.5	5.0	3.0	68.5	7407.0	3.5
SPON1	7825.0	762.5	2.9	2.9	2.8	4.5	50.8	185.3	24145.0	3.2
POSTN	8100.0	710.0	2.9	4.0	3.4	10.2	146.1	186.3	25081.3	3.4
HSP27	637.5	62.5	3.0	2.5	3.2	8.0	5.3	16.0	2001.3	2.7

QC1, high control. QC2, low control. LOB, limit of black. LLOQ, lower limit of quantitation. ULOQ, upper limit of quantification. *mean of  %CV for triplicates in all samples for each protein

### Application of the 7-plex immunoassay for the detection of UM

**Fig. 4:**
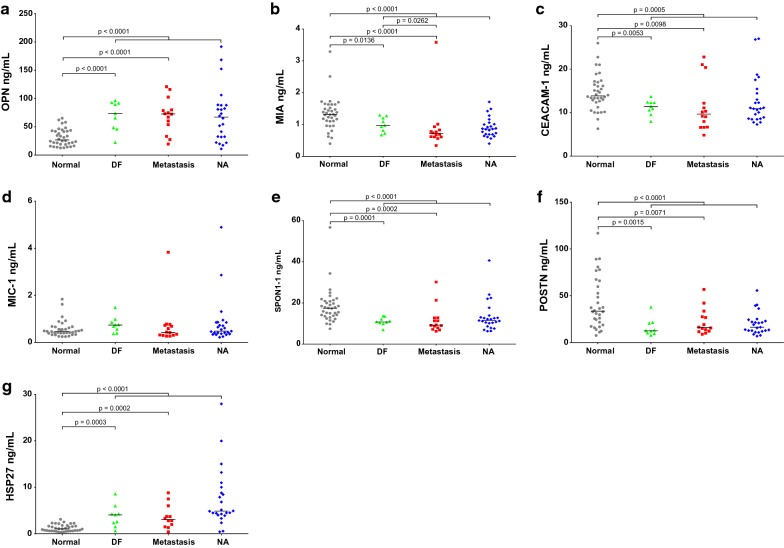
Analysis of biomarkers in sera from UM patients and healthy controls. **a**–**g** expression of OPN, MIA, CEACAM-1, MIC-1, SPON1, POSTN, and HSP27 in UM patients who either remained disease-free (DF) for at least 5 years after treatment for primary UM, patients who died with clinically overt metastases from UM, ptients in whom relavant clinicopathologic information was not available (NA), and healthy controls. Bars indicate median value

The developed 7-plex immunoassay was used to analyze the target protein levels in sera from 48 patients diagnosed with UM and 36 healthy controls (Table 1). Serum levels of OPN and HSP27 were significantly increased in UM compared to healthy controls (*p *< 0.0001), while MIC-1 was also increased in UM but not statistically significant (Fig. 4a, d, g). Meanwhile, serum levels of MIA, CEACAM-1, SPON1 and POSTN were significantly decreased in UM compared to healthy controls (*p *≤ 0.0005; Fig. 4b, c, e, f).

Serum levels of individual biomarkers were further analyzed in 9 DF (≥ 5 years) and 14 metastatic UM patients (Fig. 4a–g), although 25 patients were unevaluable as they had either shorter surveillance follow-up (< 5 years) or missing relevant clinicopathologic information. The regulation patterns of serum biomarker levels between UM and healthy controls were consistently maintained in these subgroups, when comparing either DF or metastatic UM patients to healthy controls. The serum MIA level was significantly decreased in the metastatic patient group compared to DF patient group (*p *= 0.0262), but there were no significant differences in serum levels of OPN, CEACAM-1, MIC-1, SPON1, POSTN and HSP27 between metastasis and DF groups. After excluding the highest outlier in the metastasis group (Fig. 4b, d), the downregulation of MIA in metastasis compared to DF was even more significant (*p *= 0.0083) and MIC-1 was also significantly decreased in metastasis compared to DF (*p *= 0.0354), while there were still no significant differences in the other targets between the metastasis and DF groups.

Among 38 UM patients with available information on histological type (Table 1), serum levels of individual biomarkers were further analyzed in 8 spindle cell, 25 mixed cell, and 5 epithelioid cell types (Additional file 2). The serum OPN level was significantly increased in epithelioid cell type compared to spindle cell type (*p *= 0.0451), but there were no significant differences between other subgroups. The serum HSP27 level was significantly decreased in epithelioid cell type compared to mixed cell type (*p *= 0.0298), but there were no significant differences between other subgroups. There were no significant differences in serum levels of MIA, CEACAM-1, MIC-1, SPON1 and POSTN between the three subgroups.

**Fig. 5:**
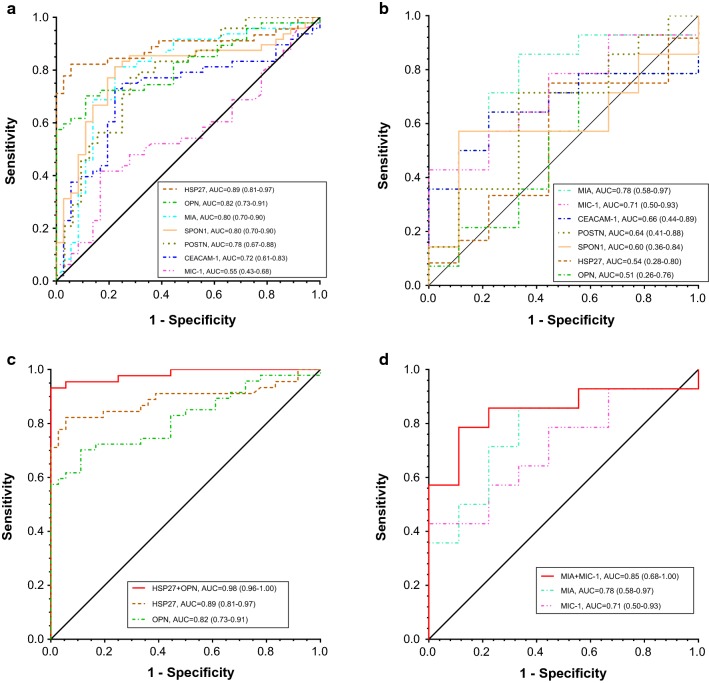
Diagnostic performance of serum biomarkers individually and combination for the detection of UM. **a** and **c** Receiver operator characteristics (ROC) curves for OPN, MIA, CEACAM-1, MIC-1, SPON1, POSTN, and HSP27 as individual markers and HSP27 and OPN combined for UM patients versus healthy controls. **b** and **d** ROC curves for OPN, MIA, CEACAM-1, MIC-1, SPON1, POSTN, and HSP27 as individual markers and MIA and MIC-1 combined for metastatic UM versus DF (≥ 5 years). The area under the curve (AUC) for each marker or panel is presented along with its 95% confidence interval in brackets

**Fig. 6:**
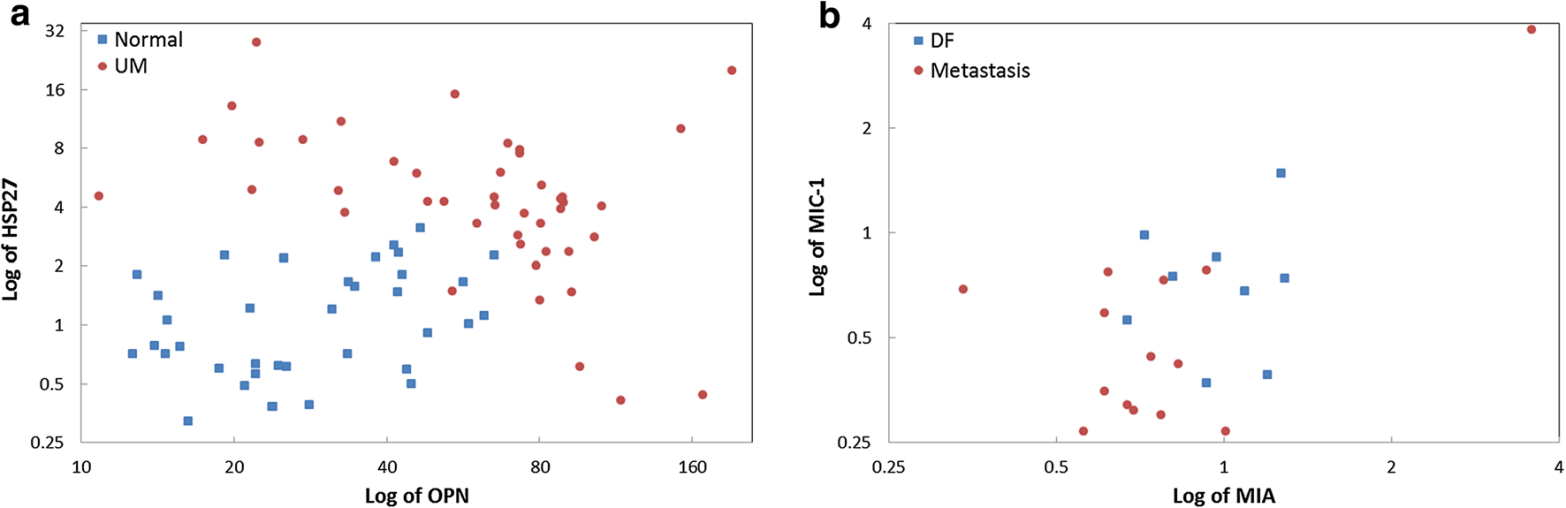
Complementary of selected serum biomarkers for the detection of UM. **a** 2D scatter plots of serum HSP27 and OPN levels for UM patients versus healthy controls. **b** 2D scatter plot of serum MIA and MIC-1 levels for metastatic UM versus DF (≥ 5 years)

Furthermore, ROC curve analysis was used to evaluate the diagnostic performance of candidate biomarkers individually and in combination for their ability to detect UM. Individually, the best biomarker (AUC, 95% CI) to separate UM from healthy controls was HSP27 [0.89, (0.81–0.97)]. Other AUCs were: OPN [0.82, (0.73–0.91)], MIA [0.80, (0.70–0.90)], SPON1 [0.80, (0.70–0.90)], POSTN [0.78, (0.67–0.88)], CEACAM-1 [0.72, (0.61–0.83)] and MIC-1 [0.55, (0.43–0.68)] (Fig. 5a). Multivariate logistic regression analysis identified a two-marker panel of HSP27 and OPN with an AUC = 0.98 (0.96–1.00), which significantly improved the individual biomarker performance of HSP27 (*p *= 0.0243) and OPN (*p *= 0.0009) in discriminating UM from healthy controls (Fig. 5c). The complementarity of HSP27 and OPN in discriminating UM from healthy controls was also demonstrated in the 2D scatter plots of serum HSP27 and OPN levels (Fig. 6a). As shown in Fig. 6a, most healthy controls plotted on the lower half (low HSP27 levels) and the left two-thirds of the scatter plot (low OPN levels); in contrast, almost all patients with UM have elevations of one or both of these two markers (top and right corner of the scatter plot).

In addition, the best individual biomarker (AUC, 95% CI) to separate metastasis from DF was MIA [0.78, (0.58–0.97)] among others, MIC-1 [0.71, (0.50–0.93)], CEACAM-1 [0.66, (0.44–0.89)], POSTN [0.64, (0.41–0.88)], SPON1 [0.60, (0.36–0.84)], HSP27 [0.54, (0.28–0.80)] and OPN [0.51, (0.26–0.76)] (Fig. 5b). Multivariate logistic regression analysis selected a two-marker panel of MIA and MIC-1 with an improved performance [0.85, (0.68–1.00)] in discriminating metastasis from DF, but yet not statistically significant due to the small sample size (Fig. 5d). The complementarity of MIA and MIC-1 in discriminating metastasis from DF was also demonstrated in the 2D scatter plot of serum MIA and MIC-1 levels (Fig. 6b).

## Discussion

In contrast to other cancers, a conventional clinical diagnosis of UM is mostly based on clinical observations by slit lamp examination, indirect ophthalmoscopy, and imaging modalities (i.e. ocular ultrasound, computed tomography, and magnetic resonance imaging) [1, 2, 41]. There are several established pathological parameters to predict the prognosis of UM patients such as tumor basal diameter, ciliary body involvement, extrascleral extension, cell type, mitotic rate, mean diameter of the ten largest nucleoli, vascular invasion, inflammation, and tumor necrosis [11, 41]. Although pathological evaluation of tumor tissues obtained by fine needle aspiration biopsies (FNABs) or enucleation can be helpful in the diagnosis and prognostic prediction of UM, it is invasive and poses certain operative risks. Recently, significant progress has been made in the understanding of the role of histopathology, cytogenetics, and gene expression patterns in predicting the risk of the metastatic UM [41]. Unfortunately, biomarkers that can be used for early detection of UM or as potential surveillance tools are still not yet available [11, 41]. In this study, a 7-plex immunoassay of OPN, MIA, CEACAM-1, MIC-1, SPON1, POSTN and HSP27 was developed in-house, validated, and applied to a set of serum samples from UM patients and healthy controls to evaluate their performance to detect UM. The assay was characterized by LOB/LLOQ, cross-reactivity, recovery, intra- and inter-assay precision; and demonstrated wide dynamic ranges for the target protein measurements that significantly correlated with their respective monoplex assays and/or commercial ELISAs. The assay demonstrated advantages over traditional ELISA and other antibody-based approaches in both multiplexing and flexibility. The assay can measure 7 candidate proteins in only 12.5 µL of serum, and allows for more candidate proteins to be added into the panel. To our knowledge, this is the first study to develop a multiplex immunoassay of human serum biomarkers for the detection of UM.

It is important to note a few general considerations for the development of a multiplex immunoassay of human serum biomarkers. First, due to the different abundances of the candidate proteins in human serum, the effective biological range of each protein must be considered to ensure the fluorescence signal falls into the dynamic range of the assay. A more sensitive assay is needed for one protein with low abundance in the 7-plex immunoassay such as MIA, while a less sensitive assay may be required for another protein which may be of high abundance in the same multiplex immunoassay such as OPN. The sensitivity of each assay may be affected by the affinity/amount of the capture antibody and the amount of capture beads used for that protein. Second, antibody characteristics such as affinity and specificity are critical for the performance of a multiplex immunoassay. All pairs of capture and detection antibodies used in this study have been tested as compatible in the sandwich ELISA for human serum samples. The majority of the capture antibodies used in this study were monoclonal antibodies which are potentially more specific than polyclonal antibodies. All of the detection antibodies except SPON1 used in this study were commercially available biotinylated antibodies. Third, the performance of the multiplex immunoassays is more analyte and sample matrix dependent compared to monoplex immunoassays [38]. Improper storage and non-optimal sample dilutions of serum samples can influence concentration measurements of some selected proteins in a complex sample matrix. It is vital to properly store serum samples at − 80 ºC prior to the analysis and avoid repeated freeze-thawing of serum samples.

The selected candidate proteins in this study have been recently assessed in UM (i.e. OPN, MIA, CEACAM-1, MIC-1, and HSP27) [9, 14–23, 30, 32, 33] and/or cutaneous melanoma [29, 35, 37] as well as other cancers [31, 34, 36] (i.e. SPON1 and POSTN) by either ELISA or immunohistochemistry or other techniques. A few research groups have proposed further evaluation of the performance of a potential combination of several serum biomarkers (i.e. OPN and MIA) for the early detection of metastatic UM [14, 17, 20]. In this study, we identified a two-marker panel of HSP27 and OPN, which significantly improved the individual biomarker performance in discriminating UM from healthy controls. The improved discrimination of a two-marker panel of MIA and MIC-1 was also observed between metastatic UM and DF, however not statistically significant due to the small sample size. Serum OPN and HSP27 levels may partially correlate with tumor histological type, but need further validation with a larger numbers of patient samples. These results highlight the value of the multiplex immunoassay in evaluating serum biomarkers that potentially complement each other in detection of UM. The simultaneous analysis of multiple proteins makes it possible to identify combinations of serum biomarkers that may have better diagnostic specificity and sensitivity than results obtained from the analysis of any single marker.

Promising serum biomarkers and their combinations for the potential early detection of UM and its hepatic metastasis should be vigorously and independently validated through a prospective longitudinal study using a larger cohort including both non-metastatic and metastatic UM patients with a longer follow-up [14, 17, 19, 20]. The performance of serum biomarkers should also be compared with the current used clinical blood tests (i.e. LFTs for hepatic metastasis), genetic features (i.e. chromosomal alterations and gene expression profiles), and pathological factors known to be associated with the prognosis of UM (i.e. tumor diameter or complex vascular patterns) [9, 11, 16, 20, 23]. Until now, the largest cohort of patient samples of UM reported in the literature was consisted of plasma samples from 449 non-metastatic UM and 54 metastatic UM for the ROC curve analysis of MIA in metastatic UM [19], while others including this study were done on relative lower numbers of total and/or metastatic patient samples [9, 14, 16–18, 20–22]. It has been pointed out that the follow-up of UM patients after the development of metastatic disease is very difficult due to the high mortality rate, and these patients are usually treated by oncologists and do not return to the eye clinic [21]. It is also very difficult to evaluate level of markers in patients without known metastasis as a true negative finding, because it is impossible to determine at what time-point micrometastases already exist when their size is still below the limit of detection with imaging modalities [19]. Previous studies have clearly demonstrated that dynamic changes in serum levels of biomarkers may be more informative than single serum levels; and individual significant increases of serum biomarkers, even within the ‘normal’ range of markers in apparently DF patients, should be followed-up immediately by imaging techniques [16, 19]. In the previous studies [14, 19–21, 23], the ‘metastatic’ and ‘non-metastatic’ subgroups were classified as the patients either with clinically proven metastases or without overt metastases at the time the plasma/serum sample was drawn, and the ‘DF’ subgroup as the patients who were disease-free for at least 10 years following treatment of the primary tumor. In contrast with these reports, in this study, 14 of 48 patients died with clinically overt metastases from UM, 9 were DF for at least 5 years, but 25 were either had shorter follow-up or relevant clinicopathologic information was not available. It was not yet clear whether metastasis already existed at the time the serum sample was drawn for these 14 UMs based on the current available clinicopathologic information. The different classification methods used for the ‘metastatic’ subgroup plus the relative short follow-up and the small sample size may contribute to the observed different alterations of serum levels of biomarkers such as MIA and MIC in the ‘metastatic’ subgroup compared to the ‘non-metastatic’ subgroup. Further validation is needed to draw a final conclusion.

## Conclusions

A magnetic bead-based multiplex immunoassay was developed demonstrating sufficient analytical performance to evaluate serum biomarkers that may complement each other in detection of UM. A prospective longitudinal study with a larger number of patient samples is needed to further validate the identified serum biomarker panels in this study. The combined use of serum biomarkers, LFTs, and imaging modalities as a screening or monitoring tool, may enable earlier clinical intervention and improve survival for patients with UM.

## Additional files

**Additional file 1. **7-plex immunoassay recombinant proteins and antibodies.

**Additional file 2. **Serum levels of individual biomarkers in uveal melanoma patients with different histological types. A-G, serum levels of OPN, MIA, CEACAM-1, MIC-1, SPON1, POSTN and HSP27 in uveal melanoma patients whose cell type was classified as spindle cell or mixed cell or epithelioid cell. Bars indicate median value.

## Abbreviations

OPN: osteopontin
CEACAM-1: carcinoembryonic antigen-related cell adhesion molecule 1
MIC-1: macrophage inhibitory cytokine-1
MIA: melanoma inhibitory activity
SPON1: spondin1
POSTN: periostin
HSP27: heat shock protein 27
