# Supplementary material for: A multiplex immunoassay of serum biomarkers for the detection of uveal melanoma
Source: Clin Proteomics. 2019 Mar 5;16:10. doi: 10.1186/s12014-019-9230-8 (PMC6399902; doi:10.1186/s12014-019-9230-8)
Supplement: Supplementary file 1 — Additional file 1. 7-plex immunoassay recombinant proteins and antibodies. [file 12014_2019_9230_MOESM1_ESM.docx]

**Additional file 1. 7-plex immunoassay recombinant proteins and antibodies.**

| **Target** | **Recombinant Protein Cat. #** |  | **Capture Antibody** | |  | **Detection Antibody** | |
| --- | --- | --- | --- | --- | --- | --- | --- |
|  |  |  | **Cat. #** | **Host** |  | **Cat. #** | **Host** |
| OPN | 1433-OP-050 |  | MAB14332 | Mouse |  | BAF1433 | Goat |
| MIA | DY2050 |  | DY2050 | Mouse |  | DY2050 | Goat |
| CEACAM-1 | DY2244 |  | DY2244 | Mouse |  | DY2244 | Goat |
| MIC-1 | DY957 |  | DY957 | Mouse |  | DY957 | Goat |
| SPON1 | 3135-SP/CF |  | AF3135 | Goat |  | AF3135* | Goat |
| POSTN | DY3548 |  | DY3548 | Mouse |  | DY3548 | Mouse |
| HSP27 | DY1580 |  | DY1580 | Goat |  | DY1580 | Rabbit |

NOTE: *, all of the recombinant proteins and antibodies were purchased from R&D Systems, except detection antibody of SPON1 was biotinylated in-house.
