# Supplementary material for: A multiplex immunoassay of serum biomarkers for the detection of uveal melanoma
Source: Clin Proteomics. 2019 Mar 5;16:10. doi: 10.1186/s12014-019-9230-8 (PMC6399902; doi:10.1186/s12014-019-9230-8)
Supplement: Supplementary file 2 — Additional file 2. Serum levels of individual biomarkers in uveal melanoma patients with different histological types. A-G, serum levels of OPN, MIA, CEACAM-1, MIC-1, SPON1, POSTN and HSP27 in uveal melanoma patients whose cell type was classified as spindle cell or mixed cell or epithelioid cell. Bars indicate median value. [file 12014_2019_9230_MOESM2_ESM.pptx]

## Slide 1
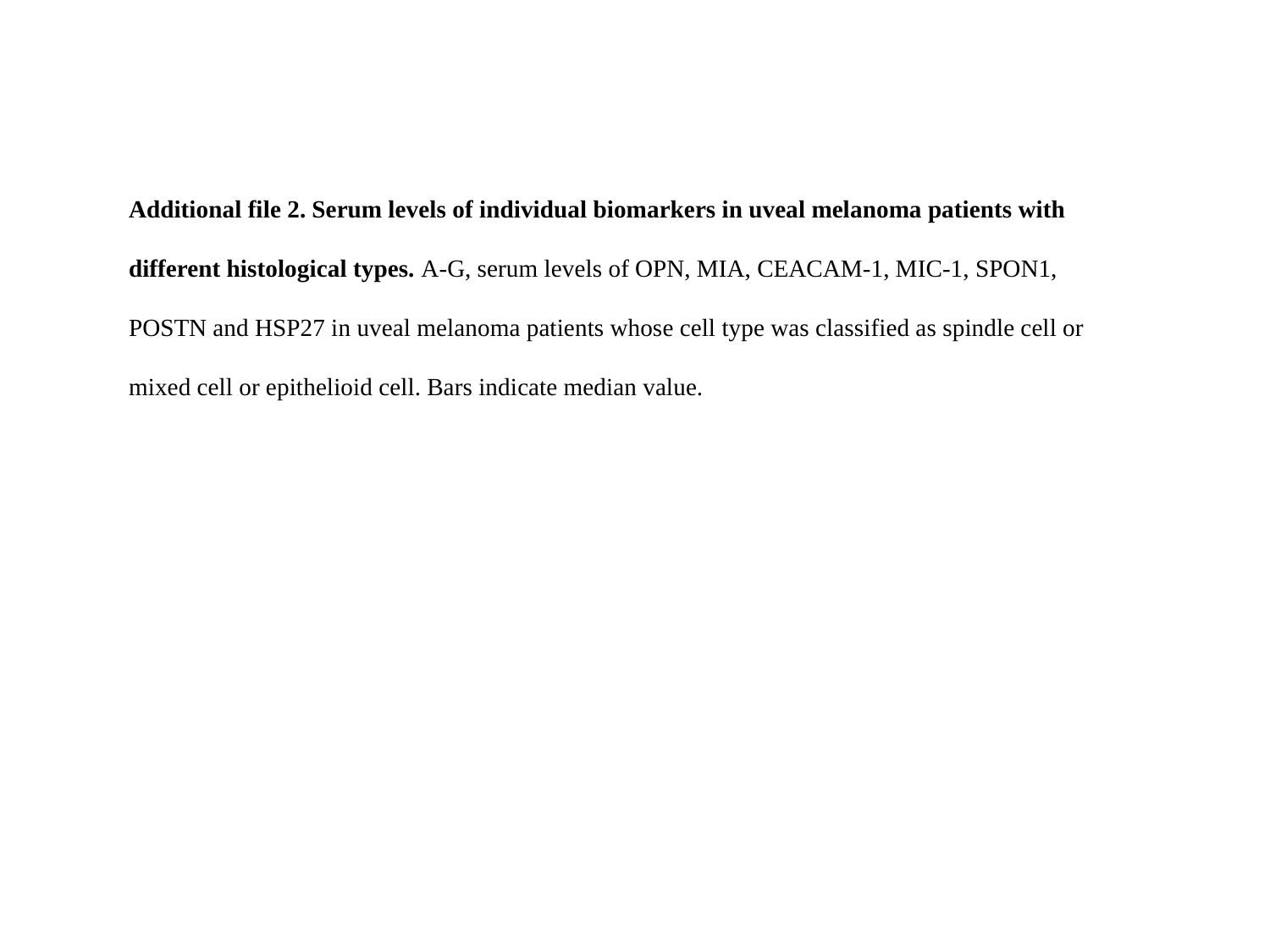

Additional file 2. Serum levels of individual biomarkers in uveal melanoma patients with different histological types. A-G, serum levels of OPN, MIA, CEACAM-1, MIC-1, SPON1, POSTN and HSP27 in uveal melanoma patients whose cell type was classified as spindle cell or mixed cell or epithelioid cell. Bars indicate median value.

## Slide 2
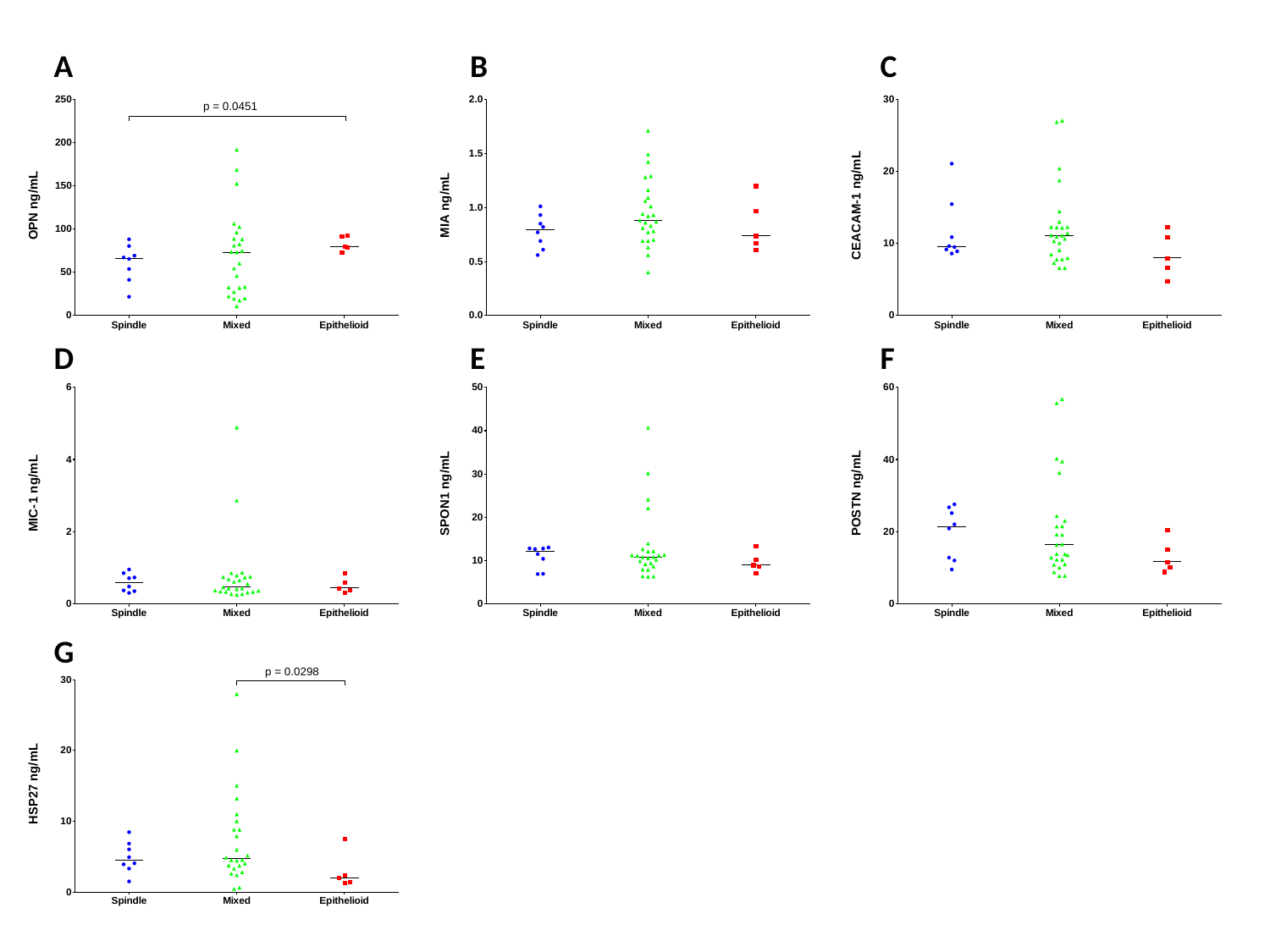

A
B
C
D
E
F
G
